# Population changes and demographic dividends

**DOI:** 10.2471/BLT.25.295004

**Published:** 2026-01-14

**Authors:** David E Bloom, Michael Kuhn, Klaus Prettner

**Affiliations:** a Harvard TH Chan School of Public Health, Huntington Avenue, Boston MA 02115, United States of America.; b Vienna Institute of Demography, Vienna, Austria.; c Vienna University of Economics and Business, Department of Economics, Vienna, Austria.

As a result of sustained declines in fertility (Fig. 1) and increases in longevity, the world’s population is undergoing major shifts in age structure (Fig. 2).^1^ Initially, fewer children lead to a reduction in the dependency ratio (that is, the ratio of the number of people who are younger than 15 years or aged 65 years and older to those aged 15–64 years), which may eventually be offset by a growing share of the population aged 65 years and older.^2^

**Fig. 1:**
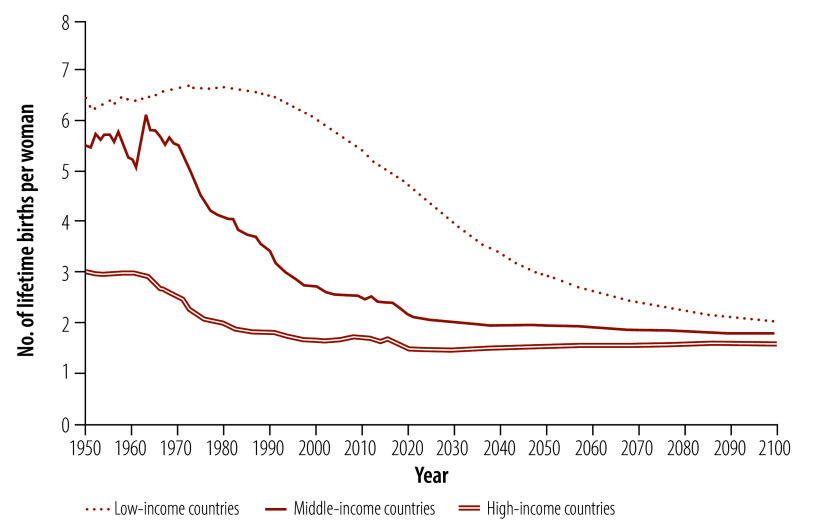
Total fertility rates, by World Bank country income group

Note: The total fertility rate is the expected number of lifetime births per woman, given age-specific fertility rates prevailing in the relevant year.

Source: United Nations Department of Economic and Social Affairs, 2024.^1^

**Fig. 2:**
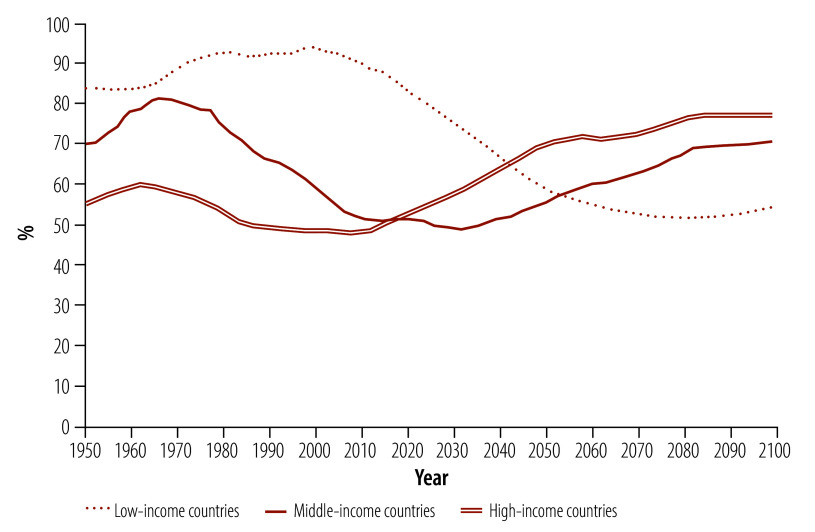
Dependency ratios, by World Bank country income group

Note: The dependency ratio is the ratio of the number of people who are younger than 15 years or aged 65 years and older to the number of people aged 15–64 years.

Source: United Nations Department of Economic and Social Affairs, 2024.^1^

The force of demography is unfolding differently across country income groups. Low-income countries will face falling dependency ratios as fertility continues to decline sharply. Middle-income countries are already experiencing relatively low dependency ratios due to past fertility declines. High-income countries face rising dependency ratios, reflecting the progression of large and increasingly long-lived cohorts to older ages.^3^^,^^4^

Demographic shifts have powerful implications for health systems, social and political stability, and economic well-being. Some of these shifts could slow economic progress, while others create opportunities for fostering economic growth, reducing poverty and increasing well-being. A comprehensive and integrated strategy of behavioural and infrastructure changes, technological innovations, institutional changes and policy advancements can mitigate adverse impacts of demographic shifts and enhance beneficial impacts.^3^

## The demographic dividend

The demographic dividend refers to accelerated economic growth and human development that can occur when declining fertility triggers a reduction in the dependency ratio. Factors that drive declining fertility include improvements in child survival, changing norms towards smaller family sizes, growing preferences for better educated children, improvements in reproductive health or even deliberate policies such as the one-child policy in China.^3^^,^^5^ Children need resources such as food, clothing, housing, education, medical care and supervision that require money or parental time. When fertility rates decline, some of these resources can be used for purposes other than child rearing, such as formal employment, expanded reach and quality of health and education systems, and development and implementation of new technologies.^6^^,^^7^ In this way, falling fertility allows countries to escape the economic and social burden of high youth dependency generated by rapid population growth. The demographic dividend can potentially boost the growth rate of income per capita by 2–3 percentage points per annum, such as in the East Asian Tiger economies in the period 1965–1995 or Ireland during the 1980s and 1990s.^6^^–^^8^ If properly managed, the demographic dividend can also be leveraged to contribute to the achievement of the sustainable development goals. A simulation study for Nigeria demonstrates how a demographic dividend embraces expansions in human capital and health across the population.^9^

Policy interventions that vary according to the stage of the demographic cycle can help realize a demographic dividend. For pre-demographic transition countries experiencing high rates of mortality and fertility, the focus would ideally be on catalysing a demographic transition, for example by promoting child survival through investments in primary health care, safe water and sanitation, immunizations and nutrition. For countries in the middle of their demographic transition, the focus would be on improving reproductive health, which tends to be associated with voluntary fertility decline. Governments can also support the transition to lower fertility by addressing unmet needs for contraception through improved knowledge of and access to modern contraception, and the promotion of girls’ education and work opportunities for women, therefore encouraging female empowerment and lower fertility.^3^^,^^5^

Despite these positive effects, fertility decline does not automatically lead to a demographic dividend, it only creates the potential for it. An actual dividend depends on complementary policies and investments in strong health and education systems, good governance, strong and appropriate infrastructure, sound macroeconomic management, carefully designed trade and migration policies, and well-functioning labour and capital markets.^3^^,^^7^ In addition, demographic dividends are likely to be larger when development strategies are well integrated across sectors. For example, insofar as childhood vaccination programmes promote child health, they also promote school attendance, cognitive function and educational attainment. As another example, programmes that enhance reproductive health reduce unwanted fertility, allow families to invest more in the health and education of their planned children, and enable parents to work and save more.^3^^,^^5^ Demographic change does not automatically determine a country’s future. Even if populations age before their countries reach high-income levels, effective policies can still generate substantial economic and social gains.

## Aiding labour-force participation

While the demographic dividend can be an important driver of economic development and poverty reduction, fertility decline eventually results in an ageing population (and possibly population decline), which generates many economic and social challenges. A comprehensive and integrated strategy of behavioural and infrastructure changes, technological innovations and economic and social policies can mitigate these challenges.^3^

### Important changes

Promoting health-seeking behaviours related to nutrition and exercise, monitoring one's own health, ensuring regular medical check-ups and promoting socialization are especially important to the health, activity levels, productivity and well-being of older people.^10^ These practices are consistent with the World Health Organization’s framework for implementing a life course approach to health.^11^ Improved availability of health care, and appropriate infrastructure such as parks and senior centres that enable or encourage healthy activities, can support such behavioural changes.

In addition, economic and social policies can expand the labour force by enabling later retirement. Linking the statutory retirement age to increases in life expectancy is often proposed as a potential approach. However, such policies tend to be unpopular, and they ignore the fact that some individuals may have had demanding jobs and have health-related conditions that prevent them from working longer. Softer reforms such as supporting phased retirement, increasing flexibility in the retirement system to allow those who wish to work longer to do so, and removing financial disincentives to work at older ages can substantially increase the number of economically active individuals.^3^ Strengthening lifelong learning programmes and mid-career retraining while improving occupational health are important actions governments can take for older people to remain productive. These enabling policies are well complemented by adequate health, disability and unemployment insurance for those unable to keep pace with rapid technical change or having to leave the labour force for health reasons.

Another critical aspect of ensuring high employment involves increasing women’s participation in the labour force, particularly by promoting parental leave schemes that are compatible with a relatively smooth return to work, and by expanding access to affordable, high-quality childcare and early childhood education.^12^

### Technological innovations

Continued investment in technological innovation is essential for addressing the challenges of demographic shifts. In terms of health innovation for an ageing population, investing in novel pharmaceuticals and medical devices; continuing development and widespread use of vaccines for disease prevention; investing in diagnostics, including wearables for monitoring blood glucose, hypertension, balance and other clinically important parameters; and expanding telemedicine platforms help ensure that individuals can age with dignity and enjoy active leisure well into advanced ages.

Beyond health, technological progress can also make a substantial economic contribution in ageing societies. Investments in research and development and the adoption of labour-saving or labour-enhancing technologies such as advanced robotics, 3D (three-dimensional) printing and artificial intelligence can help offset slower labour-force growth.^13^ The productivity impacts of such investments are amplified when they are combined with investments in education and population health.^3^

### Population-oriented policies

Policy measures can help mitigate the effects of population ageing. For example, targeted immigration of skilled workers can help alleviate labour shortages in key sectors, provided complementary investments in education and labour market integration accompany such immigration. However, political resistance to high immigration as well as potential negative effects of (skilled) labour outflows associated with out-migration need to be considered as potential constraints.

Family policies that promote the compatibility of child rearing and employment may inhibit or reverse declining fertility.^12^ However, increasing fertility may come at the short-term cost of creating a reverse demographic dividend until the larger cohorts begin to enter the labour force, which is typically about 20 years after the fertility increase.^3^

In addition, it will be necessary for public and private entities in the health sector and beyond to expand the capacity and quality of long-term care systems (both formal and informal), with attention to the supply of trained care workers and issues of affordability and equitable access.^14^

Integrated policies are more likely to succeed than isolated reforms. For example, policies enabling later retirement may be more effective, feasible and compatible with maintaining social well-being when older workers are healthy and skilled. Similarly, migration policies are most effective when combined with robust integration strategies.

## Conclusion

A key goal of countries facing population ageing and population decline is to mitigate the corresponding health, social and economic challenges. Achieving this goal requires careful attention to the design and implementation of targeted approaches to promoting female labour force participation, later retirement and selective migration; boosting productivity growth through better education and health; and making greater investments in research and development and labour-saving technologies such as industrial robots and artificial intelligence. Particularly in low-income countries, a key goal remains ensuring the emergence of a generous demographic dividend and sustaining it by investing wisely in general and reproductive health throughout the life cycle, as well as in education, infrastructure, institutional development and technology adoption. In all countries, ensuring that the enacted policies are consistent with one another and leverage the potent complementarities among health, education, retirement and technological progress is essential.
